# A case of metastasectomy following multiple courses of radionuclide therapy for distant metastatic pheochromocytoma

**DOI:** 10.1210/jcemcr/luag307

**Published:** 2026-10-05

**Authors:** Naoki Wada, Satoshi Kawata, Yoshinari Obata, Yoshito Tomimaru, Junji Kozawa, Iichiro Shimomura

**Affiliations:** Department of Metabolic Medicine, Graduate School of Medicine, The University of Osaka, Suita, Osaka 565-0871, Japan; Department of Metabolic Medicine, Graduate School of Medicine, The University of Osaka, Suita, Osaka 565-0871, Japan; Department of Metabolic Medicine, Graduate School of Medicine, The University of Osaka, Suita, Osaka 565-0871, Japan; Department of Gastroenterological Surgery, Graduate School of Medicine, The University of Osaka, Suita, Osaka 565-0871, Japan; Department of Metabolic Medicine, Graduate School of Medicine, The University of Osaka, Suita, Osaka 565-0871, Japan; Department of Diabetes Care Medicine, Graduate School of Medicine, The University of Osaka, Suita, Osaka 565-0871, Japan; Department of Metabolic Medicine, Graduate School of Medicine, The University of Osaka, Suita, Osaka 565-0871, Japan

**Keywords:** pheochromocytoma, radionuclide therapy, surgery, ^131^I-MIBG, ^177^Lu-DOTATATE

## Abstract

Pheochromocytomas and paragangliomas (PPGLs) are neuroendocrine tumors derived from chromaffin cells that produce catecholamines. The primary treatment for PPGLs is surgical resection, and even in cases with distant metastases, metastasectomy may be considered. Other therapeutic options for distant metastatic disease include radionuclide therapy, such as ^131^I-metaiodobenzylguanidine (MIBG) and ^177^Lu-1,4,7,10-tetraazacyclododecane-1,4,7,10-tetraacetic acid-(Tyr^3^)-octreotate (DOTATATE). However, clear evidence is lacking regarding which patients with distant metastases are the best candidates for metastasectomy or radionuclide therapy. Herein, we report a case of a 58-year-old man who underwent resection of a left pheochromocytoma 10 years prior and was admitted for treatment of recurrent metastases to the liver and peritoneum. Sequential ^131^I-MIBG and ^177^Lu-DOTATATE therapies were administered, which contributed to disease control and reduction of catecholamine levels but did not achieve a complete response. Subsequently, all metastatic lesions were successfully resected, leading to normalization of catecholamine secretion. This case suggests that metastasectomy may be preferable to radionuclide therapy in carefully selected patients with limited, resectable metastatic disease, despite the presence of distant and multiple metastases. Furthermore, it highlights the importance of periodic reassessment of resectability in patients with distant metastases, even when radionuclide therapy is initially selected, and consideration of metastasectomy once complete resection becomes feasible.

## Introduction

Pheochromocytomas and paragangliomas (PPGLs) are rare neuroendocrine tumors derived from chromaffin cells [1, 2]. Most of these tumors hypersecrete catecholamines; therefore, patients often present with hypertension, and if untreated, cardiovascular morbidity and mortality are high [3]. Surgical resection is the primary treatment for PPGLs. The National Comprehensive Cancer Network Clinical Practice Guidelines recommend surgical resection whenever feasible [4]. Even in cases with distant metastases, metastasectomy may be considered. Several cases of metastatic pheochromocytomas undergoing metastasectomy have been reported [5-7]. Other therapeutic options for distant metastatic disease include chemotherapy and radionuclide therapy. Regarding radionuclide therapy, ^131^I-metaiodobenzylguanidine (MIBG) therapy is considered for ^123^I-MIBG–positive tumors, while peptide receptor radionuclide therapy with ^177^Lu-1,4,7,10-tetraazacyclododecane-1,4,7,10-tetraacetic acid-(Tyr^3^)-octreotate (DOTATATE) is an option for tumors expressing somatostatin receptors [8, 9]. Radionuclide therapy has shown promise in controlling hypertension and reducing tumor burden [10, 11]. However, clear evidence is lacking regarding which patients with distant metastases are the best candidates for metastasectomy or radionuclide therapy. Additionally, to the best of our knowledge, few reports have described metastasectomy following radionuclide therapy in which the effects of both therapeutic approaches could be evaluated within the same patient. Herein, we present a case of recurrent pheochromocytoma with distant metastases after resection of the primary lesion. The patient was treated with sequential ^131^I-MIBG and ^177^Lu-DOTATATE therapies, which contributed to disease control and reduced catecholamine levels but did not achieve a complete response. He subsequently underwent metastasectomy, resulting in normalization of catecholamine levels. This case may help guide the selection of metastasectomy and radionuclide therapy for individual patients with distant metastatic PPGL.

## Case presentation

A 58-year-old man was admitted to our hospital for treatment of recurrent metastatic pheochromocytoma. At age 45, he visited a clinic for headaches and was found to have systolic blood pressure exceeding 200 mmHg. His headaches worsened, and he developed hyperhidrosis, prompting him to seek medical attention again at age 48. Laboratory tests revealed elevated urinary normetanephrine (8.45 mg/day [International System of Units (SI): 46.1 µmol/day], ref: 0.10-0.28 mg/day [SI: 0.5-1.5 µmol/day]) and plasma norepinephrine (2813 pg/mL [SI: 16.62 nmol/L], ref: 150-570 pg/mL [SI: 0.89-3.37 nmol/L]). Abdominal computed tomography (CT) revealed an approximately 8-cm left adrenal tumor, and ^123^I-MIBG scintigraphy showed intratumoral uptake, leading to a diagnosis of pheochromocytoma. He subsequently underwent open left adrenalectomy, which improved his blood pressure and symptoms. At age 52, his plasma norepinephrine level was within the normal range (244 pg/mL [SI: 1.44 nmol/L]), and no recurrence was observed. He subsequently discontinued follow-up visits. At age 57, his headaches recurred, and upon reevaluation at his previous hospital, ^123^I-MIBG scintigraphy revealed intratumoral uptake in the liver and the peritoneum on the greater curvature of the stomach. These findings were consistent with recurrent metastatic pheochromocytoma. He was then referred to our hospital for further evaluation and treatment.

## Diagnostic assessment

On admission, his height was 163.4 cm, he weighed 63.2 kg, and his body mass index was 23.7 kg/m^2^. His family history included the sudden death of his younger sister at age 20 from an unknown cause. His blood pressure remained stable at 108/71 mmHg while taking doxazosin 1 mg. Physical examination revealed a midline abdominal surgical scar; however, no other abnormalities were observed. Laboratory tests revealed a mildly elevated blood glucose level (125 mg/dL [SI: 6.9 mmol/L], ref: 65-110 mg/dL [SI: 3.6-6.1 mmol/L]), a slightly elevated hemoglobin A1c level (6.3%, ref: 4.6-6.2%), and an increased triglyceride level (206 mg/dL [SI: 2.32 mmol/L], ref: 50-150 mg/dL [SI: 0.56-1.69 mmol/L]). However, electrolyte levels and complete blood counts were normal. His plasma norepinephrine level was elevated at 4200 pg/mL (SI: 24.8 nmol/L), and plasma-free normetanephrine was also elevated at 1994.6 pg/mL (SI: 10.89 nmol/L, ref: <506 pg/mL [SI: <2.76 nmol/L]). His plasma epinephrine level was <10 pg/mL (SI: <54.6 pmol/L, ref: ≤170 pg/mL [SI: ≤928.0 pmol/L]), and his plasma-free metanephrine level was normal at 16.4 pg/mL (SI: 0.08 nmol/L, ref: <130 pg/mL [SI: <0.66 nmol/L]). Similarly, urinary tests showed elevated urinary norepinephrine at 2159 µg/day (SI: 12 762 nmol/day, ref: 29.2-118 µg/day [SI: 173-697 nmol/day]) and elevated urinary normetanephrine at 5.4 mg/day (SI: 29.5 µmol/day). Urinary epinephrine was 3.57 µg/day (SI: 19.5 nmol/day, ref: 1.1-22.5 µg/day [SI: 6.0-123 nmol/day]), and urinary metanephrine was 0.07 mg/day (SI: 0.355 µmol/day, ref: 0.05-0.20 mg/day [SI: 0.254-1.01 µmol/day]), both within normal limits. Computed tomography revealed liver nodules measuring 34 mm in S7 and 36 mm in S6, a 28-mm peritoneal nodule along the greater curvature of the stomach, and a 4-mm nodule in the right upper lobe of the lung (Fig. 1A–1D). Both ^123^I-MIBG and ^111^In-pentetreotide scintigraphy showed radiotracer uptake in the liver and peritoneal nodules (Fig. 1E–1G and 1I–1K), indicating postoperative distant metastases of pheochromocytoma. No radiotracer uptake was observed in the pulmonary nodule; however, pulmonary metastasis could not be excluded (Fig. 1H and 1L).

**Figure 1:**
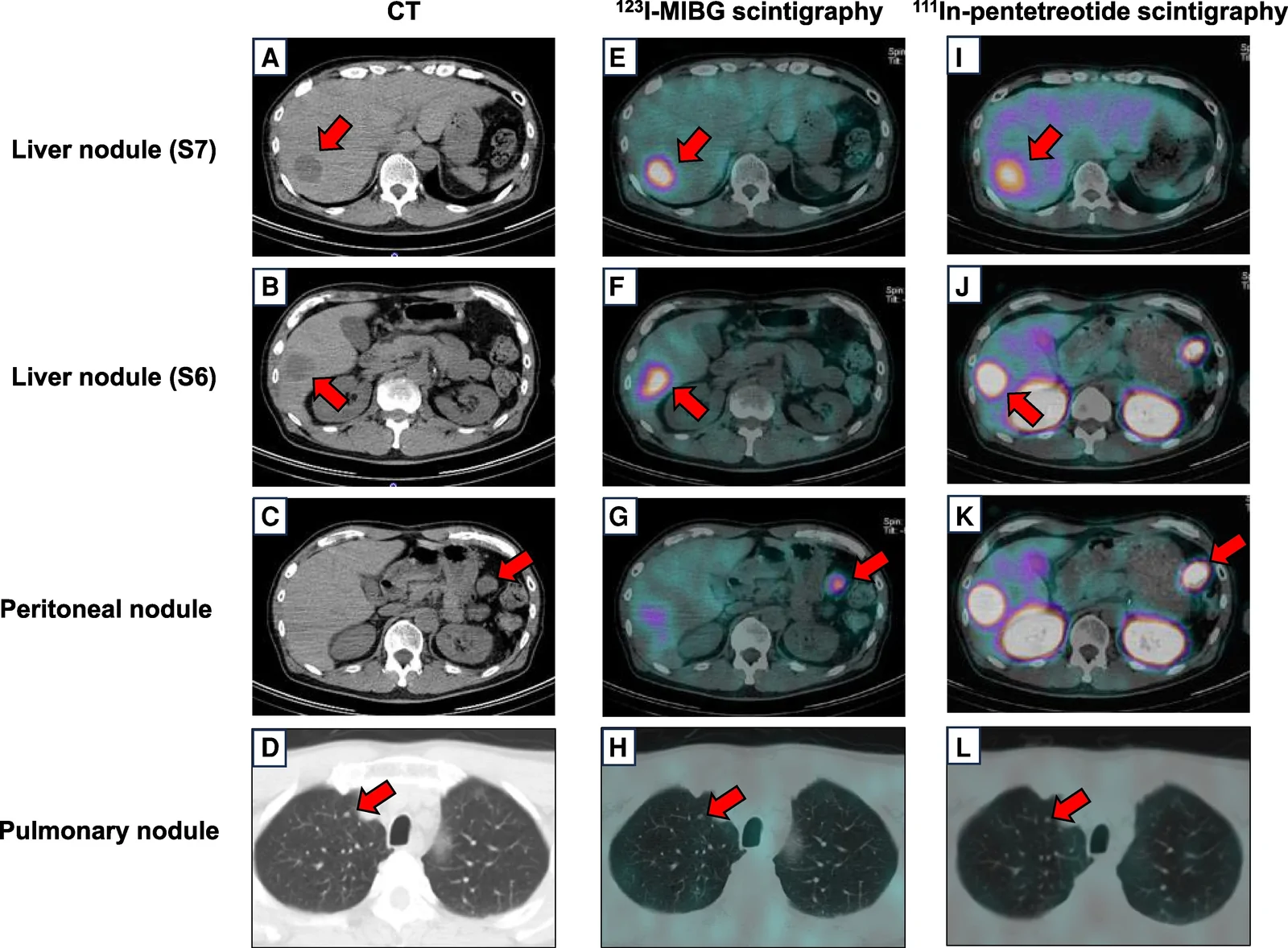
Imaging of liver, peritoneal, and pulmonary nodules. (A, E, I) Liver nodule in the right posterior superior segment (S7). (B, F, J) Liver nodule in the right posterior inferior segment (S6). (C, G, K) Peritoneal nodule. (D, H, L) Pulmonary nodule. (A–D) Noncontrast computed tomography (CT). (E–H) ^123^I-metaiodobenzylguanidine (MIBG) scintigraphy. (I–L) ^111^In-pentetreotide scintigraphy.

## Treatment

Metastasectomy for the abdominal lesions was considered and discussed with the patient. However, he declined surgery because of concerns about enlargement of the pulmonary nodule and early development of new metastatic lesions after surgery. Therefore, a shared decision was made to proceed with radionuclide therapy. As both somatostatin receptor and ^123^I-MIBG scintigraphy demonstrated comparable uptake, he was deemed eligible for treatment with either ^131^I-MIBG or ^177^Lu-DOTATATE. Initial low-dose ^131^I-MIBG therapy (7.4 GBq) was administered because efficacy of single-dose therapy has been reported in Japanese cohorts [12]. However, no reduction in catecholamine levels or tumor size was observed 4 months after treatment, with the best radiological response being stable disease (SD) according to the Response Evaluation Criteria in Solid Tumors (RECIST) version 1.1 (Fig. 2A and 2B–2D). Given the absence of both biochemical and radiological response, the therapeutic strategy was then switched to ^177^Lu-DOTATATE (7.4 GBq), and 4 cycles were administered at 2-month intervals. The cumulative administered activity was 7.93 GBq for ^131^I-MIBG and 27.79 GBq for ^177^Lu-DOTATATE. Following treatment, plasma catecholamine levels decreased to approximately half of baseline, and his headaches resolved. The liver metastases showed no significant change in size, whereas the peritoneal lesion decreased in size; however, the overall best radiological response was SD (Fig. 2A and 2F–2H). As the pulmonary nodule remained stable without radiotracer uptake throughout radionuclide therapy, pulmonary metastasis was considered unlikely (Fig. 2E and 2I). Furthermore, there was no evidence of new metastatic lesions. Therefore, complete resection of all metastatic lesions was deemed feasible. After further shared decision-making, he underwent robot-assisted laparoscopic partial hepatectomy 10 months after completing ^177^Lu-DOTATATE therapy. Preoperatively, the doxazosin dose was gradually increased to 16 mg, maintaining blood pressure at 110 to 130/65 to 90 mmHg. During surgery, blood pressure remained stable at 100 to 130/45 to 55 mmHg without significant fluctuations. Although a peritoneal metastasectomy was planned, the peritoneal lesion could not be identified intraoperatively and was therefore not resected. No postoperative complications occurred.

**Figure 2:**
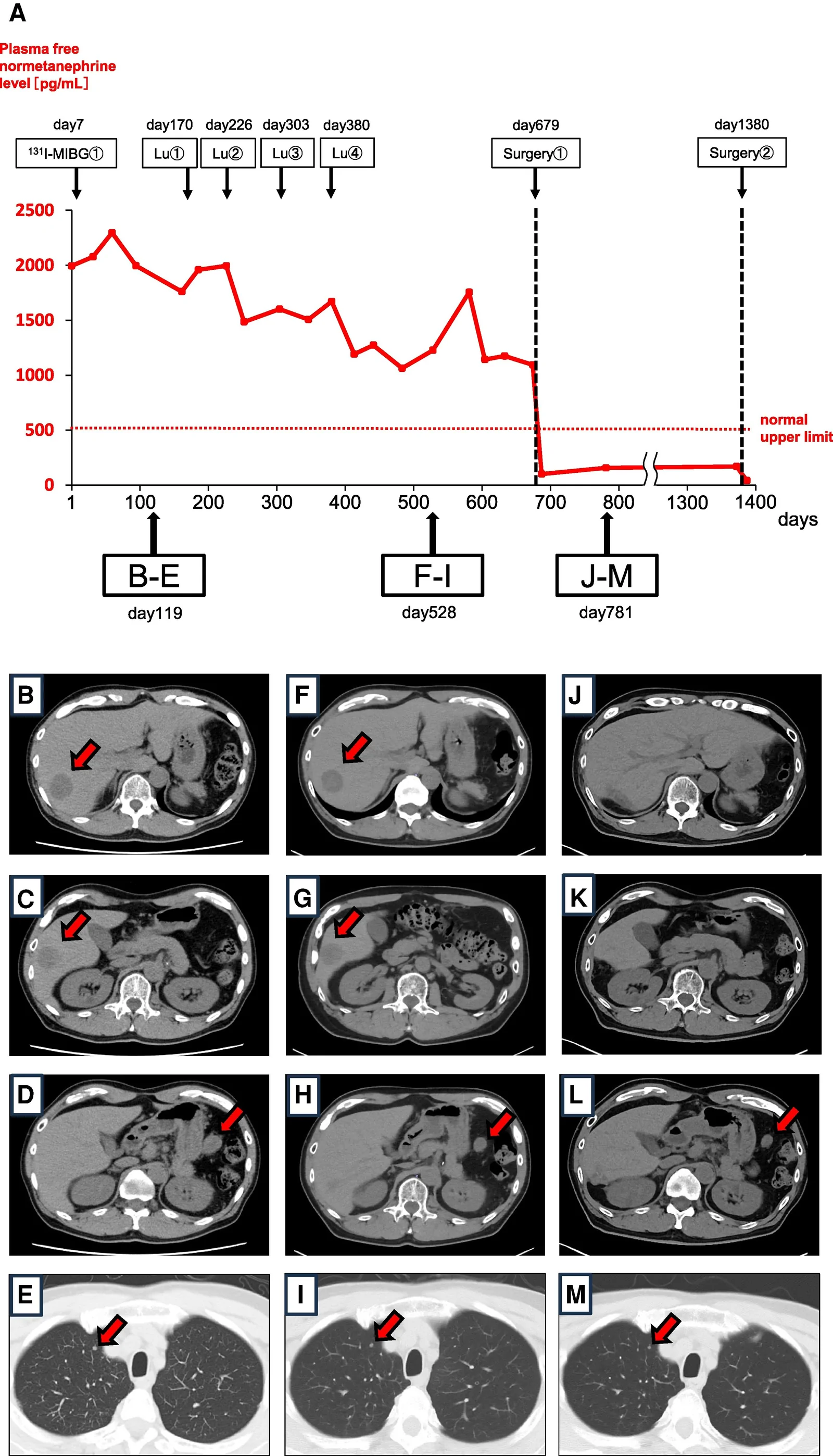
(A) Chart showing the treatment course and changes in plasma-free normetanephrine levels. (B–M) Imaging of liver, peritoneal, and pulmonary nodules during the clinical course. (B–E) Four months after ^131^I-metaiodobenzylguanidine (MIBG) therapy. (F–I) Five months after 4 cycles of ^177^Lu-DOTATATE therapy. (J–M) Three months after surgery.

## Outcome and follow-up

On gross examination, a well-demarcated, white-colored tumor measuring 30 mm in diameter was identified in S7 of the liver, along with a dark brown tumor with partially black areas measuring 35 mm in S6 (Fig. 3A). Microscopic examination revealed a Zellballen architecture of tumor cells surrounded by capillaries (Fig. 3B and 3C) and immunopositivity for chromogranin and synaptophysin (Fig. 3D and 3E), consistent with metastatic pheochromocytomas. The tumor cells were negative for succinate dehydrogenase B (SDHB). The surgical margin was negative. Following surgery, residual peritoneal lesions remained detectable (Fig. 2J–2L); however, plasma catecholamine levels normalized and remained within the normal range during the 21-month follow-up period (Fig. 2A). The size of the peritoneal lesion had slightly increased; therefore, open surgical tumor resection was performed, achieving complete resection. Postoperatively, plasma catecholamine levels decreased further (Fig. 2A). During follow-up, the pulmonary nodule remained stable (Fig. 2M). Given the family history of sudden death in a sibling, metastatic disease, and loss of SDHB immunostaining, an underlying germline variant in an SDH-related gene was suspected. However, the patient declined genetic testing.

**Figure 3:**
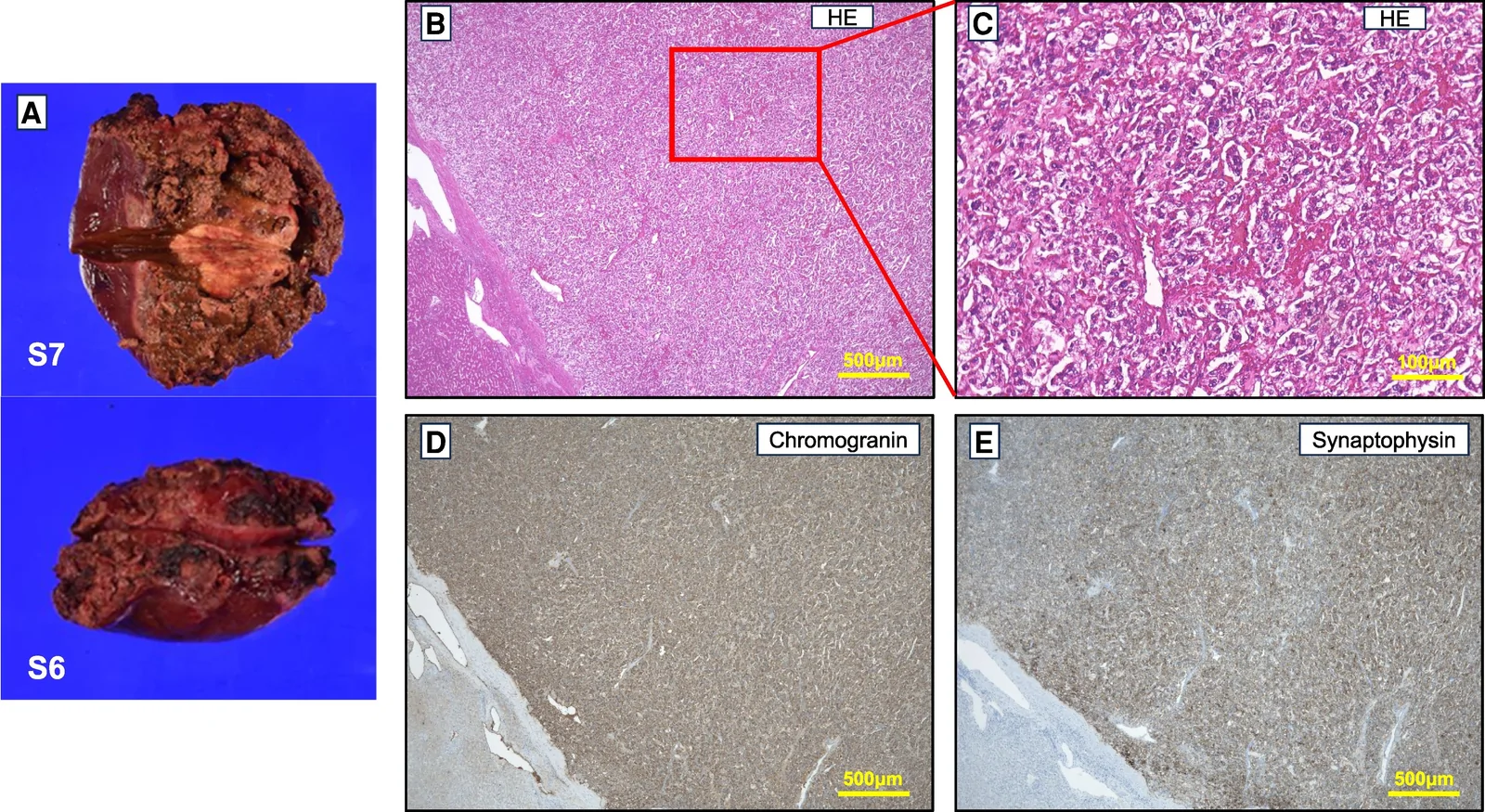
Cut surfaces and light microscopic findings of the resected tumor. (A) Cut surfaces of the resected tumor in S6 and S7 of the liver. Histological analysis of the S6 liver metastasis using hematoxylin and eosin staining (B, C) and immunohistochemical staining for chromogranin (D) and synaptophysin (E).

## Discussion

We present a case of metastatic pheochromocytoma initially treated with 2 radionuclide therapies. These treatments contributed to tumor progression control and partial reduction of catecholamine levels; however, a radiological or biochemical complete response was not achieved. Subsequently, all metastatic lesions were successfully resected, resulting in normalization of catecholamine secretion. To the best of our knowledge, this is a rare case of distant metastatic PPGL in which metastasectomy was performed following radionuclide therapy, allowing assessment of the effects of each therapeutic approach within the same patient. This case provides insight into the expected benefits of each approach and their appropriate selection.

Radionuclide therapy has demonstrated radiological and biochemical efficacy in metastatic PPGL. The patient received low-dose ^131^I-MIBG (7.4 GBq) and ^177^Lu-DOTATATE therapies. According to the European Association of Nuclear Medicine guidelines, ^131^I-MIBG is typically administered at 3.7 to 11.1 GBq per cycle [13]. However, in Japan, owing to strict regulatory limits on the use of radiopharmaceuticals, low-dose protocols (5.55-7.4 GBq) administered every 3 to 4 months for multiple cycles are common [14]. Regarding radiological response assessed by CT or magnetic resonance imaging using the RECIST version 1.1, the disease control rate (SD or better) has been reported to be as high as 90.7% [15]. In contrast, ^177^Lu-DOTATATE is typically administered at 7.4 GBq every 2 months, for a maximum of 4 cycles, consistent with international practice [11]. In one study, 87.5% of patients achieved SD or better [16]. With respect to biochemical response, a clinical trial of a single low-dose ^131^I-MIBG therapy (7.4 GBq) reported a ≥50% reduction in urinary catecholamines in 23.5% of patients, indicating a modest biochemical effect even with a single administration [12]. In contrast, a study of ^177^Lu-DOTATATE therapy (7-8 GBq for up to 4 cycles) reported a ≥50% reduction in urinary catecholamines in 64% of patients, highlighting its potential efficacy with repeated treatment [11]. These findings suggest that both radionuclide therapies are effective in controlling tumor progression and reducing catecholamine secretion in metastatic PPGL. However, a radiological or biochemical complete response is rarely achieved with either treatment.

A previous study has suggested that surgical resection of the primary lesion in metastatic PPGL may provide clinical benefits, even when complete resection is not possible [17]. Additionally, despite the limited evidence, metastasectomy has been suggested to provide a potential survival advantage [18]. Hepatectomy for liver metastases, including debulking surgery, has also been reported to reduce catecholamine levels and relieve symptoms [19]. Accordingly, both radionuclide therapy and metastasectomy may be useful in treating distant metastatic PPGL. However, clear evidence is lacking regarding which patients with distant metastases are the best candidates for each approach. In this case, both radionuclide therapies likely contributed to disease control and prevented the development of new lesions. Furthermore, although the initial ^131^I-MIBG therapy did not reduce catecholamine levels, subsequent ^177^Lu-DOTATATE therapy reduced plasma-free normetanephrine levels to approximately half of baseline. Nevertheless, metastasectomy was ultimately required to achieve a biochemical complete response. Since high tumor burden and elevated catecholamine levels are known risk factors for intraoperative complications [20], preoperative radionuclide therapy may help reduce catecholamine levels and lower surgical risk. However, this observation alone does not support the routine use of radionuclide therapy prior to metastasectomy. Rather, this case suggests that metastasectomy may be preferable to radionuclide therapy in carefully selected patients with limited, resectable metastatic disease, even when the metastases are distant and multiple.

This case report has several limitations. First, the long-term outcomes remain unknown, and continued follow-up is required. Second, as a single case, further studies are needed to clarify the optimal use of radionuclide therapy and metastasectomy in patients with distant metastatic PPGL.

In summary, we present a case of recurrent metastatic pheochromocytoma in which metastasectomy following radionuclide therapy successfully normalized catecholamine levels. This case suggests that metastasectomy may be preferable to radionuclide therapy in carefully selected patients with limited, resectable metastatic disease. Furthermore, it highlights the importance of periodic reassessment of resectability in patients with distant metastases, even when radionuclide therapy is initially selected, and consideration of metastasectomy once complete resection becomes feasible.

## Learning points

- Both radionuclide therapy and metastasectomy are potential treatment options for distant metastatic PPGL.
- Metastasectomy may be preferable to radionuclide therapy in carefully selected patients with limited, resectable metastatic disease.
- Surgical resectability of metastatic lesions should be periodically reassessed, even when radionuclide therapy is initially selected.

## Data Availability

Original data generated and analyzed for this case report are included in this published article.
